# Comparative EST analysis provides insights into the basal aquatic fungus *Blastocladiella emersonii*

**DOI:** 10.1186/1471-2164-7-177

**Published:** 2006-07-12

**Authors:** Karina F Ribichich, Raphaela C Georg, Suely L Gomes

**Affiliations:** 1 Departamento de Bioquímica, Instituto de Química, Universidade de São Paulo, Av. Prof. Lineu Prestes 748, São Paulo, SP 05508-000, Brazil

## Abstract

**Background:**

*Blastocladiella emersonii *is an aquatic fungus of the Chytridiomycete class, which is at the base of the fungal phylogenetic tree. In this sense, some ancestral characteristics of fungi and animals or fungi and plants could have been retained in this aquatic fungus and lost in members of late-diverging fungal species. To identify in *B. emersonii *sequences associated with these ancestral characteristics two approaches were followed: (1) a large-scale comparative analysis between putative unigene sequences (*uniseqs*) from *B. emersonii *and three databases constructed *ad hoc *with fungal proteins, animal proteins and plant unigenes deposited in Genbank, and (2) a pairwise comparison between *B. emersonii *full-length cDNA sequences and their putative orthologues in the ascomycete *Neurospora crassa *and the basidiomycete *Ustilago maydis*.

**Results:**

Comparative analyses of *B. emersonii uniseqs *with fungi, animal and plant databases through the two approaches mentioned above produced 166 *B. emersonii *sequences, which were identified as putatively absent from other fungi or not previously described. Through these approaches we found: (1) possible orthologues of genes previously identified as specific to animals and/or plants, and (2) genes conserved in fungi, but with a large difference in divergence rate in *B. emersonii*. Among these sequences, we observed cDNAs encoding enzymes from coenzyme B_12_-dependent propionyl-CoA pathway, a metabolic route not previously described in fungi, and validated their expression in Northern blots.

**Conclusion:**

Using two different approaches involving comparative sequence analyses, we could identify sequences from the early-diverging fungus *B. emersonii *previously considered specific to animals or plants, and highly divergent sequences from the same fungus relative to other fungi.

## Background

Since the sequencing of the first complete fungal genome, the budding yeast *Saccharomyces cerevisiae *[1], fungal genomics and the specific area of comparative genome analysis in fungi have experienced a recent but impressive advance. Following sequencing of the genomes of two other ascomycetes, *Schyzosaccharomyces pombe *and *Neurospora crassa *[2,3], efforts have focused on species throughout the fungal kingdom that represent diverse scientific interests. In this sense, genomes from plant and human pathogenic basidiomycetes have been sequenced [4,5] and there are drafts or genome projects in progress of other fungi. Likewise, the sequencing of one zygomycete genome has been completed and there are two chytrid genome projects underway (see [6] for an overview of fungal genome sequencing projects).

Expressed sequence tag (EST) data from fungi, even though less numerous than genome sequences, are also showing to be useful to specific and diverse aims, such as mapping previously characterized genes [7], investigation of patterns of fungal genome evolution [8], prediction of novel genes [9], prediction of pathogenicity determinants [10], identification of disease-related sequences [11], improvement of functional assignments [12], and identification of alternatively spliced mRNA species [13].

Recently, we reported a sequencing program of nearly 17,000 ESTs corresponding to different developmental stages of *Blastocladiella emersonii *life cycle, an early diverging fungus that belongs to the Chytridiomycete class [14,15]. Approximately 52% of the *uniseqs *presented similarity to sequences deposited in public data banks. Interestingly, several of these ESTs revealed similarity with known genes not previously reported in fungi, and which had been recognized as animal or plant specific proteins. Despite the fact that a consensus phylogenetic tree seems to resolve fungi and animals as sister groups [16], we wondered if some ancestral characteristics of fungi and animals or of fungi and plants could have been retained in this basal fungus and have been lost or become highly divergent in members of the late-branching group of fungi.

Our previous study provided the functional identification of putative unique transcripts based on sequence comparison, and contributed to linking *in silico *expression profile data with previous information about biological processes occurring throughout the fungal life cycle [14]. In this sense, the survey increased the knowledge about this interesting biological model. However, our approach did not provide a direct link between expressed sequences in *B. emersonii *and gene expression in major groups, like animals and plants.

In the present work, we carried out a large-scale comparative analysis of *B. emersonii *ESTs against protein and transcript sequences of fungi, animals and plants, using databases constructed *ad hoc*. Our goals were to identify putative orthologues in *B. emersonii *of genes previously classified as specific to animals and/or plants, as well as to find *B. emersonii *sequences common to fungi but which have evolved at a lower rate in this chytrid than in late-diverging fungi. Based on our results, we discuss possible relationships between expressed sequences and structures and/or biological processes occurring in animals and/or plants and *B. emersonii*, including a metabolic pathway previously reported only in animals and bacteria.

## Results

### *B. emersonii*-animal shared sequences

To uncover sequences shared by *B. emersonii *and animals, we carried out a comparison between *B. emersonii *ESTs and an animal database constructed *ad hoc*, and assigned a putative identification to these sequences (see Methods section below). We then classified *B. emersonii *sequences that matched with animal data (named *B. emersonii*-animal shared sequences) as follows: hits only found in animals; hits found in animals and protists (including flagellated and ciliated organisms and green algae not filtered as plants on purpose, some hits also included bacteria); hits only found in animals (when using an Evalue ≤ 10^-5 ^as cut-off) but with protein family members also described in plants and/or fungi; hits found in animals and bacteria (Table 1).

**Figure 1:**
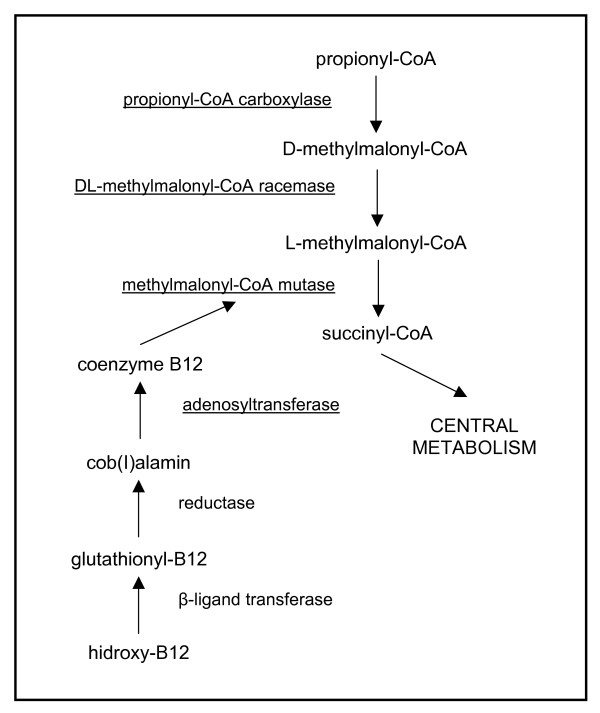
**Scheme of the pathway of cobalamin-dependent propionyl-CoA metabolism**. Enzymes mentioned in the text and in Table 1 are underlined.

**Figure 2:**
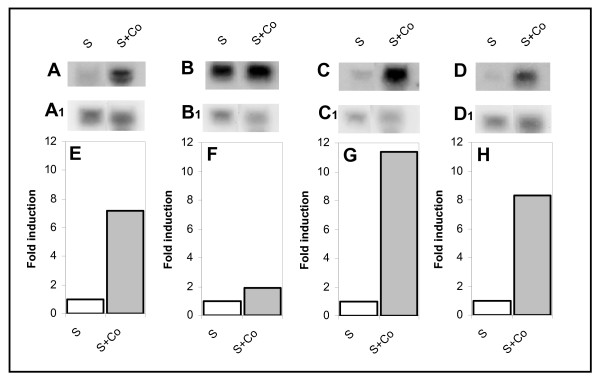
**Northern blot analysis of *B. emersonii *genes encoding enzymes involved in propionyl-CoA metabolism**. A, E. Propionyl-CoA carboxylase; B, F. DL-methylmalonyl-CoA-racemase; C, G. Methylmalonyl-CoA mutase; D, H. ATP:Cob(I)alamin adenosyltransferase. The RNA blots were also hybridized with a probe of the *hsp70-3 *gene, which is not induced by CoCl, as a control (A_1_-D_1_). Data from densitometry scanning of the hybridization bands is shown in panels E-H. The values were normalized using the *hsp70-3 *bands as a control. S = total RNA isolated from cells after 60 min of sporulation; S + Co = total RNA isolated from cells after 60 min of sporulation in the presence of 100 μM CoCl.

As the most important result, matches only with animal proteins revealed two consensus sequences encoding enzymes involved in coenzyme B_12_-dependent propionyl-CoA metabolism: DL-methylmalonyl-CoA racemase (EC 5.1.99.1) and methylmalonyl-CoA mutase (EC 5.4.99.2). We also found ESTs encoding the alpha and beta chains of propionyl-CoA carboxylase (EC 6.4.1.3), the enzyme that catalyzes the first step of this metabolic route (Table 1 and Figure 1). These enzymes give the capacity to metabolise propionate through propionyl-CoA and methylmalonyl-CoA in the TCA cycle and they seem to be present in most animal species and prokaryotes [17,18] but there are no sequences or activities described in fungi. Furthermore, methylmalonyl-CoA mutase needs adenosylcobalamin (coenzyme B_12_) as a cofactor and we wondered whether sequences encoding enzymes involved in biosynthesis of coenzyme B_12 _would be expressed in *B. emersonii*. Interestingly, we found another assembled sequence, among the matches with animal and bacteria proteins, encoding an ATP:Cob(I)alamin adenosyltransferase (EC 2.5.1.17), the enzyme that catalyses the last step of coenzyme B_12 _biosynthesis. Afterwards, we proceeded to do an experimental validation of the expression of these sequences in the fungus. As shown in Northern blot assays (Figure 2A–H), these genes are expressed during *B. emersonii *sporulation. In addition, as cobalt is necessary for the pathway to function, we also evaluated expression of these genes in cells exposed to cobalt and all four genes appeared to be induced by this cation (Figure 2E,H).

Moreover, approximately 10% of the hits obtained through this approach were related to flagella and significant alignments appeared with animals and *Chlamydomonas reinhardti*, suggesting that these sequences are conserved among different organisms. Finally, 13% of the matches included animals and euglenozoos, as *Trypanosoma cruzi*, and 10% included the ciliated *Tetrahymena thermophila*. A small proportion of two hits revealed sequences that retrieved significant matches only with animal proteins, but which belong to protein families with members among plants and fungi. However, both of them are not well characterized.

### *B. emersonii*-plant shared sequences

After removal of contaminants, as carried out for *B. emersonii*-animal shared sequences, we classified the hits found against the plant database (*B. emersonii*-plant shared sequences) as follows: hits only found in plants; hits found in plants and protists; hits only found in plants but with protein family members also described in animals and/or fungi; hits found in plants and bacteria (Table 2).

The first important difference observed in *B. emersonii*-animal and *B. emersonii*-plant sequence comparison was the number of *uniseqs *with matches in each group: the number of matches with plants was one fifth of the number obtained with animal proteins (20 vs. 105). However, some noteworthy information could be obtained. Three putative protein receptors: a phytosulfokine receptor, an ethylene receptor CS_ETR2 and a protein kinase receptor (the first two mentioned in [14]), which are plant receptors not previously found among fungi, were found in *B. emersonii*.

On the other hand, two *B. emersonii *assembled sequences presented significant matches only with plants but encode proteins that belong to families with members in animals and fungi. One of them encodes a putative Isp4 protein, which represents a family of transporters of small oligopeptides (OPT family), initially characterized only in three different species of yeast [19-21]. A set of related proteins from *Arabidopsis thaliana*, characterized as oligopeptide transporters, was later described as an outgroup to the yeast set by neighbor joining analysis [22]. The *B. emersonii *assembled sequence aligns with a significant score only to sequences of the plant OPT family and not to the fungal sequences. Nevertheless, although the assembled sequence presents the conserved regions characteristic of the protein family of both animals and fungi, the region of alignment comprises less than 60% of the total length of the best matching sequence. Thus, the assignment of a putative function for the protein should be taken with caution.

The other assembled sequence matched with a member of the syntaxin family of soluble N-ethylmaleimide-sensitive factor adaptor protein receptors (SNAREs) superfamily, which is known to play an important role in the fusion of transport vesicles with specific organelles [23]. In a general sense, animals and plants have syntaxins that are orthologues to one of the yeast syntaxins. However, whereas some classes of yeast and mammalian syntaxin genes appear to be absent in *Arabidopsis*, its genome presents syntaxin gene families not found in other eukaryotes [24]. The SYP7 family (with three members) does not appear to have an ortholog among yeast or animal syntaxins, and this group may be unique to plants. The *B. emersonii *assembled sequence mentioned above matched with a putative syntaxin 71, a member of SYP7 family. We also looked for *B. emersonii *ESTs encoding other syntaxin family members and found representatives for all except one (SYP8) of the families categorized according to Sanderfoot *et al*. [24](Table 3).

### *B. emersonii*-animal-plant shared sequences

Following the same procedure carried out for the two previous analyses, we classified the hits found against the animal and plant databases (*B. emersonii*-animal-plant shared sequences) as follows: hits found in animals and plants; hits found in animals, plants and protists (some hits also included bacteria); hits found in animals and plants but with protein family members also described in fungi; hits found in animals, plants and bacteria (some hits also included protists) (Table 4).

A noteworthy identification was a putative urocanate hydratase, urocanase or imidazolone-propionate hydrolase (EC 4.2.1.49), the second enzyme involved in the catabolism of histidine by conversion of this amino acid to glutamate [25]. We also found an EST encoding an imidazolonepropionase (EC 3.5.2.7), among the matches with animal and bacteria proteins, the third enzyme in the same pathway. The first enzyme of the pathway is the histidase or histidine-ammonia lyase, which converts histidine into urocanate, the substrate of the urocanase. Urocanase has been found in bacteria, in the liver of mammals, in the land plant white clover, and also in *Chlamydomonas reinhardtii *(see [26] and ref. therein; [27]). This activity is probably present in protists and other plants as *Medicago sativa*, according to sequences deposited in Genbank protein database. In bacteria, the degradation of histidine to glutamate provides the organism with a source of carbon and nitrogen (see [28] and ref. therein). Fungi apparently lack urocanase activity, as revealed by the absence of genes encoding the enzyme in fungal sequence resources. The enzyme activity has been specifically searched in *Aspergillus nidulans *[28]. This fungus synthesizes an active histidase enzyme but cannot use histidine as the sole carbon source, which has been attributable to the lack of an active urocanase; histidine is quantitatively converted to urocanate, which accumulates in the extracellular medium.

Among the sequences recovered, we also observed a type C fructose-bisphosphate aldolase (FBA). This type of enzyme belongs to the Class I aldolase family, whose members have been observed mainly in higher eukaryotes. Fungi FBAs belong to the Class II aldolase family, presenting little similarity with proteins from Class I (Rutter, 1964 in [29]). In addition, we did not find another FBA in *B. emersonii *transcript database.

Three different putative proteins from *B. emersonii*, originated from full length cDNA sequences, do not have orthologues in other fungi but are found in animals and plants, and present similarity with the MtN3 family of proteins according to Pfam database. Although the molecular function of the proteins that compose this family is unknown, they are almost certainly transmembrane proteins. One of the *B. emersonii *putative proteins contains six transmembrane regions and one MtN3 domain [BeDB: BeAS884], another presents seven transmembrane regions and two MtN3 domains [BeDB: BeAS315], and the third one contains five transmembrane regions and one MtN3 domain [BeDB: BeZSPN18F02], according to the Interpro program package.

We also identified a novel sequence not previously identified in fungi: a singlet encoding a gamma-SNAP protein (soluble N-ethylmaleimide-sensitive factor-attachment protein). Whereas alpha-SNAP homologues have been identified in yeast, plant, mollusk and insect cells, gamma-SNAP homologues have been found only in mammals, plants and more recently *Dictyostelium discoideum *[30]. In addition, a cDNA encoding an alpha-SNAP homologue was also observed in *B. emersonii *database, showing that this fungus has the two different types of SNAP proteins.

Finally, among *B. emersonii*-animal, *B. emersonii*-plant and *B. emersonii*-animal-plant shared sequences, the highest percentage of matches (approximately 65%) was achieved for sequences encoding proteins classified in unknown processes. In fact, the functional characterization of these sequences remains one of the most important challenges in post-transcriptome research.

### Sequence divergence comparison between *B. emersonii *and *N. crassa *or *U. maydis*

**Figure 3:**
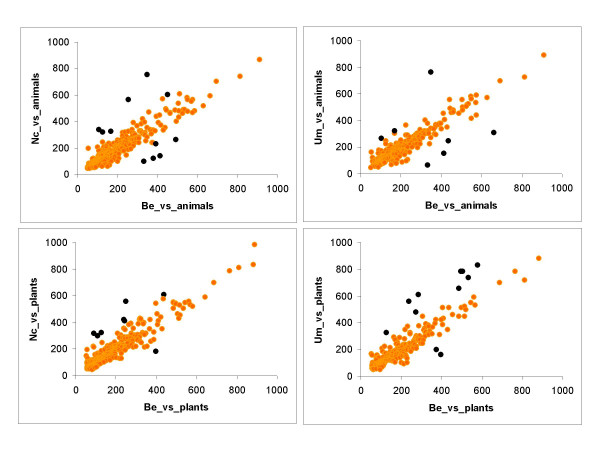
**Pairwise score comparison between fungal orthologues and animal and plant sequences**. Black dots on the plots represent score-pairs with a difference in *bit score *equal or higher than 150.

We also carried out a comparative analysis to identify *B. emersonii *putative genes with a higher degree of similarity to animal or plant genes than to their fungal counterparts. The S' values, obtained for pairs of putative orthologues from *N. crassa*/*U. maydis *and *B. emersonii*, were plotted with their best matches in animal or plant sequences. Pairs of hits with highest differences in S' values in two or more comparisons were chosen for further investigation (Figure 3). Four apparently divergent sequences were identified and three of these were, unexpectedly, more divergent in *B. emersonii *than in *N. crassa *and *U. maydis *(Table 5). None of the four sequences appeared related by biological process, function or localization. In addition, two of them, encoding a putative Rbj-like protein and an elongation factor 1 alpha long form, do not have clear orthologous relationships.

Rbj-like or Rjl proteins are members of Ras-related GTP-binding proteins. Rjl proteins have recently been identified as a new family, independent of the Rab family, to which they were initially linked [31]. There is no evidence for a role for these proteins in other organisms, except chordates [31]. As no Rjl sequences were identified in other fungi, we looked for family signatures in *B. emersonii *deduced protein sequence, and we also checked *N. crassa*, animal and plant data, which had been collected as orthologues.

In the putative protein from *B. emersonii*, four of the family characteristics identified by Nepomuceno-Silva *et al*. [31] were observed: 1) the substitution of the canonical glutamine residue in the third GTP binding domain; 2) the alteration of the DTAGQE motif to DMAGDR (it is the first motif with E to R substitution); 3) the percent identity with other Rjl proteins (between 37 and 40%), with only one exception; 4) the absence of a prenylation motif. Using a hidden Markov Model [32,33], a signal peptide prediction was made but with low probability (58 %), and no signal anchor was predicted, as is expected for Rjl proteins.

The apparent *N. crassa *ortholog [Genbank: EAA33910] and its best matches among animal and plant data were GTPases from the Rab family. Likewise, the best match of *B. emersonii *Rjl in the plant database was a Rab protein [Genbank: AK062838], in agreement with the absence of Rjl records in land plants. In contrast, when compared with animal data, *B. emersonii *Rjl aligned better with an Rbj protein from *Tetraodon nigroviridis *[Genbank: DAA01331], which is the expected Rjl ortholog in chordates.

The elongation factor 1-α (EF1-α), a core member of the protein biosynthesis machinery, is ubiquitous in eukaryotes and in prokaryotes, where it is named EF-Tu [34]. *B. emersonii *presents an EF-like or EFL protein, which is different from the canonical EF1-α identified in the majority of the organisms [14]. Due to this fact, we expected to sample the *B. emersonii *divergent sequence encoding the EFL protein during this procedure. Although EFL and EF1-α probably perform similar roles, they are clearly different proteins, and EFL proteins form a completely separated branch in molecular phylogenies. Moreover, taxa genomes with EF1-α lack EFL, suggesting that EFL has replaced eEF-1 α several times independently [34]. However to our surprise, a first data processing revealed no significant S' difference (ΔS') between the pair *B. emersonii *EFL/plant protein match, and *N. crassa*/*U. maydis *EF1-α/plant protein matches, even though these two fungi present the canonical form of EF1-α. The explanation for this unexpected result is that *Oryza sativa *genome apparently contains two different genes [Genbank: AK110624 and AK107366], one that matched with the sequence encoding *B. emersonii *EFL, and another that matched with the fungal *N*. crassa and *U. maydis *EF1-α. In addition, *O. sativa *genome also presents a third gene encoding the canonical EF1-α [Genbank: AK103738] usually found in plants. The first two rice sequences do not seem to be contaminant products, as no positive results were obtained using blastn against Genbank non-redundant or dbEST databases. Altogether, *O. sativa *genome seems to contain three genes encoding divergent EF1-α. Whether or not all three sequences actually represent rice genes requires clarification.

Another assembled sequence shown to be divergent in *B. emersonii *encodes a mitochondrial ADP/ATP translocase. This translocase, also known as a mitochondrial adenine nucleotide translocator (ANT), catalyses the exchange of ATP and ADP between mitochondria and cytosol, and seems to participate in mitochondrial events that control cell death (see [35] and ref. therein.) The divergence of *B. emersonii *sequence was only observed when the comparison was carried out against the plant database. A molecular phylogeny based on neighbor-joining distances following a Poisson model resolved *B. emersonii *sequence in a branch separate from other fungi, which diverged closer to plant than to animal counterparts (data not shown). This tree is in agreement with the differences revealed through local alignments. Curiously, B. *emersonii *branch was shared with three other sequences from evolutionarily distant organisms: *Dictyostelium discoideum *[RefSeq:XM_642074], *Phytophtora infestans *[Genbank: AAN31467] and *Oryza sativa *[Genbank: AK060330]. In addition, we identified three more *O. sativa *unigenes together with the above sequence: the canonical plant sequence [RefSeq:XP_467495], one that branched with fungi [Genbank: AK110815], and another very similar to a *U. maydis *sequence [Genbank: AK108179], which remained unresolved as its putative fungal ortholog.

Finally, we found sequences encoding a putative 2-amino-3-carboxymuconate-6-semialdehyde decarboxylase or ACMSD (EC 4.1.1.45), an enzyme involved in the tryptophan-niacin pathway in eukaryotes. There are few eukaryotic and even fewer prokaryotic sequences known. Fungal sequences are poorly characterized, and they are apparently absent from plants. Moreover, we could observe that some bacterial sequences diverged with the eukaryotic counterparts in a molecular phylogeny constructed with the same method used for the putative ANT (data not shown). This observation suggests the possibility of lateral gene transfer, as proposed by Muraki *et al*. [36]. *B. emersonii *deduced protein appears to have the two conserved motifs described for ACMSD proteins by the same authors, even though the motifs possess clear differences from those found in fungal homologues.

## Discussion

We carried out a comprehensive comparative EST analysis that identified one hundred and sixty-six expressed sequences from the aquatic fungus *B. emersonii *encoding putative proteins not previously reported in other fungi.

Among the ESTs with significant similarity to animal sequences, we found assembled sequences encoding enzymes involved in coenzyme B_12_-dependent propionyl-CoA metabolism. Propionate, the second most abundant fatty acid in soil, is formed by fermentative processes from carbohydrates and several amino acids [37]. Propionate is converted to propionyl-CoA, which is also formed by oxidation of odd-chain fatty acids and several amino acids, and then converted to succinyl-CoA that then enters the central metabolism. This pathway is used in diverse metabolic processes and homologues of intervening enzymes were found within archaeal, bacterial and eukaryal genomes, but not in plants or fungi. In mammals, this route is employed in the catabolism of valine, isoleucine, methionine, threonine, thymine, cholesterol, as well as odd-chain fatty acids [38], and defects in some of the enzymes involved lead to the rare but severe inherited disease methylmalonyl aciduria [17].

Propionate is generally toxic to fungi and bacteria, and this is the reason why it is widely used as a preservative [39]. Despite its toxicity, many bacteria and fungi are able to use propionate as carbon and energy sources under aerobic conditions, using an alternative pathway to that mentioned above, the "methyl citrate cycle" that catalyses the oxidation of propionate to pyruvate.

Fungi such as *S. cerevisiae *and *Aspergillus nidulans *seem to lack cobalamin-dependent functions and therefore cannot use the methyl-malonyl-CoA pathway [18]. Leadley *et al*. [18] suggested that the maintenance of this pathway in proto-eukaryotes would have meant a high evolutionary cost, due to the need to preserve also the enzymes capable of producing coenzyme B_12_, and at the same time, the existence of other pathways for propionate utilization may have superceded the selective pressure for preserving this metabolic route.

The question is why *B. emersonii *would express coenzyme B_12_-dependent enzymes under the conditions tested. The presence in archaea, eubacteria and animals of coenzyme B_12 _and coenzyme B_12_-dependent enzymes seems to indicate that the conservation of these functions is important to diverse processes and this principle can also be applied to fungi. Despite the absence of genes encoding cobalamin-dependent proteins and enzymes of coenzyme B_12 _biosynthesis in the fungal genomes sequenced, pathways involving this coenzyme could be active under conditions not frequently tested in other fungi whose genomes have not been sequenced yet. In fact, some of *B. emersonii *ESTs encoding these enzymes were isolated from a cDNA library constructed with mRNA isolated from cells exposed to high concentrations of cadmium. Differently from cadmium, several transition metals, such as cobalt, play a role as catalysts in a variety of enzymatic reactions. These metals, which are normally useful to the cells, can be toxic when in excess. Thus, many molecular mechanisms for cell detoxification have been developed. Some of these mechanisms are promiscuous, being responsible for detoxification of more than one of these heavy metals [40]. In this sense, we cannot rule out the possibility that some *B. emersonii *genes induced by exposure to cadmium could be involved in cobalt metabolism.

Our analysis has also shown eleven sequences associated to flagella-related proteins expressed in *B. emersonii *and animals, nine of them also present in green algae. The absence of these sequences from fungi and plant databases was expected, as a consequence of the bias in the most investigated species, which mainly belong to late-diverging fungi and land plants. Thus, *B. emersonii *could be a good model to study processes related to flagella structure and movement, probably contributing to the characterization of differences between animals and other flagellated cells.

Comparison of *B. emersonii *ESTs with plant sequences revealed two assembled sequences with high similarity to genes found only in plants, but encoding proteins that belong to families with members also in animals and fungi: a putative Isp4 protein (an oligopeptide transporter) and a putative syntaxin 71 (a member of SYP7 family of protein receptors). Oligopeptides can be used as source of amino acids, nitrogen and carbon, and their transporters have been documented in bacteria, fungi and plants. The identification of multiple OPTs in *Arabidopsis*, with tissue-specific expression patterns, supports the idea of different functional roles for these transporters, e. g., regulators of hormone activity in hormone-peptide conjugates [22]. There is also evidence indicating that members of other peptide transporter family, the PTR, have a role in plant growth and development. Thus, it is possible that the putative OPT found in *B. emersonii *has a specific function, different from those described in other fungi, as regulation of growth and differentiation.

In this same context, we can include the matches of *B. emersonii *ESTs with two plant receptors associated with the control of proliferation and development in plants. Even though the alignments extend over a conserved region in the plant sequences, domains characteristic of the assignments do not overlap. Consequently, *B. emersonii *proteins could be involved in completely different processes. Further studies will be necessary to clarify this hypothesis.

The syntaxin family of proteins is well represented in *B. emersonii *transcriptome. We found representatives for all except one (SYP8) of the defined families [24] (Table 3). The group included syntaxin 71, a member of SYP7 family, which seems exclusive of plants. Such broad representation suggests that syntaxin 71 could have specific functions in *B. emersonii*, perhaps related to functions developed in plants.

Members of the syntaxin family are known to play an important role in the fusion of transport vesicles with specific organelles [23], and specifically SYP7 proteins seem to be involved in transport between the ER and the Golgi apparatus [41]. Interestingly, membrane transport and vesicle rearrangement have critical importance during the sporulation stage of *B. emersonii *life cycle [42], and several ESTs related to this function were exclusively isolated from sporulating cells (see [43] GO:0006886 intracellular protein transport), which includes the EST encoding the possible syntaxin 71.

*B. emersonii*-animal-plant common sequences included an urocanase, an enzyme with no records in sequenced fungal genomes and which could be indicative of *B. emersonii*'s ability to use histidine as a carbon source. The presence of sequences encoding enzymes possibly involved in the catabolism of valine, isoleucine, methionine and threonine (such as the enzymes that are active in coenzyme B12-dependent propionyl-CoA metabolism), and enzymes possibly involved in the catabolism of histidine, suggest that *B. emersonii *metabolism might be directed towards amino acid catabolism, as a source of carbon. Early studies in chytrids indicated distinct roles for some amino acids, other than serving as nitrogen source or protein building blocks. For instance, certain amino acids have been shown to be effective in initiating growth on sugars different from glucose, such as mannose and fructose, in *Allomyces macrogynus *cultures, presumably supplying both carbon and nitrogen sources [44].

An assembled sequence encoding a FBA type C, a member of class I FBA, was also found in our analyses. FBAs are divided into two non-related protein classes: Class I FBA, not found in fungi but with widespread distribution in other eukaryotes and also found in prokaryotes, and Class II FBA, identified mainly in eubacteria and also in eukaryotes, including fungi [29,45]. Although the scattered taxonomic distribution of FBA classes does not have a consensual evolutionary explanation yet, gene duplication events and replacement of one paralog by the other are events that could have occurred. For instance, there is some evidence for the existence of an ancestral class II aldolase, from the endosymbiosis with a cyanobacterium, which could have been replaced by a class I aldolase in red and green algae, as well as in higher plants [29,46]. Class II FBA genes of ascomycetes are also of eubacterial origin, and probably consequence of endosymbiosis with mitochondria ancestors [47]. Thus, a gene replacement event such as the proposed for red and green algae and land plants could similarly be proposed for the origin of *B. emersonii *FBA gene.

Even more interesting than the presence of a member of class I FBA in *B. emersonii*, could be the type observed, the C type, which is supposed to have evolved after divergence of the B type [48]. In fact, no B type FBA sequences were observed among *B. emersonii *ESTs. One possible explanation would be that the C type FBA could have replaced the B type. Another explanation, perhaps the simplest one, is that the B type sequence was not found among *B. emersonii *sequenced ESTs, but the gene is present in the genome. However, why both types of FBAs would be expressed in *B. emersonii *is not clear yet. In vertebrates, Class I comprises three types of isozymes expressed in different tissues: aldolase A (muscle type, also expressed in brain), B (liver type) and C (brain type). As described for other enzymes of the glycolytic pathway, aldolases A and C display activities different from that observed during glucose metabolism, as they regulate the stability of the light neurofilament mRNA through their ribonuclease activity [49]. A specialized function for the C type aldolase in *B. emersonii *should not be ruled out.

In addition, two putative genes encoding the alpha and gamma-SNAPs were observed in *B. emersonii*. Until now, no gamma-SNAPs have been described in fungi, *B. emersonii *being the first fungus in which this gene has been identified. However, the presence of both alpha and gamma-SNAPs in eukaryotic cells seems to be the rule, with fungi being the exception, considering that five phylogenetically distant species are known to possess both alpha and gamma-SNAP: *D. melanogaster*, *B. taurus*, *H. sapiens*, *A. thaliana *and *D. discoideum *[30]. The protein alpha-SNAP is essential for membrane traffic because it allows efficient NSF/SNARE interaction. Instead of this direct function, gamma-SNAP could have a regulatory role in membrane fusion. It was also suggested a role for gamma-SNAP in mitochondrial dynamics, contributing as an adaptor in the attachment of mitochondria to the cytoskeleton [50]. Our results in *B. emersonii *indicate that *D. discoideum *is not the only simple eukaryote containing both alpha and gamma-SNAPs.

As a second approach to discover non-typical fungal genes in *B. emersonii*, we carried out a comparative analysis with the expressed sequences of this aquatic fungus and other fungal sequences. We intended to be conservative at the time of selection and very few sequences were identified. Likewise, several difficulties arose due to the complexity of dealing with large multigene families. Indeed, two of the four selected sequences initially collected were not orthologues, and the relationship between the other two is not evident, but we decided to include these sequences in our analysis because the information extracted was also relevant. In fact, even though the sequences encoding the Rjl protein were not reported in fungi or plants, we did not detect them among the 105 *B. emersonii*-animal-shared sequences selected in our first approach. The divergence found for the other three cases (EF1α, ADP/ATP translocase and aminocarboxymuconate semialdehyde decarboxylase) is also noteworthy, because it could reflect high evolutionary rates, gene duplication and replacement (as suggested for EF1α in [34]), gene conversion, or horizontal gene transfer from prokaryotes to eukaryotes or among eukaryotes, which seems to be more common that previously thought (see [31]).

This collection of selected *B. emersonii *assembled sequences represents the result of approaches that use comparative EST analyses to address differences and similarities between chytrids and other eukaryotes (other fungi, animals and plants). The results of such analyses will probably suffer modifications when more fungal sequences are available. Specifically, other chytrid and zygomycete sequences will contribute to define retained, lost and divergent genes. Moreover, at least part of the borderline sequences (with an Evalue between 10^-3 ^and 10^-5 ^against *ad hoc *fungal database), which could be true divergent homologues, could constitute a group of interest to help understand phylogenetic relationships among fungi.

## Conclusion

Through two different approaches involving comparative sequence analyses, and using computational tools and manual revision, we identified 162 protein-coding sequences from *B. emersonii *previously described in animals (such as coenzyme B_12_-dependent propionyl-CoA pathway members, and proteins related to flagella structure or movement), in plants (such as protein receptors, a putative member of small olipeptide transporter, and a SYP7 family member of syntaxins), and in animals and plants (such as an urocanase, a fructose-bisphosphate aldolase (FBA) type C, members of the MtN3 family and a gamma-SNAP representative). We also found 4 sequences from *B. emersonii*, which were identified in a fungal sequence comparison as not found or highly divergent from other fungal species: a Rbj-like protein (similar to animal proteins), an EF-like protein (dispersely distributed in taxa, already described in [14]), an ADP/ATP translocase (similar neither to plant nor to animal sequences) and a 2-amino-3-carboxymuconate-6-semialdehyde decarboxylase (different from fungal sequences, poorly characterized). When the selected ESTs were classified according to the biological processes in which they could be involved, cell growth and maintenance, signal transduction and metabolism resulted as the biological processes most represented. Some sequences selected were expected, based on the knowledge about chytrids, like those associated to specific structures not found in other fungi (e. g., flagellar-associated ESTs). Thus, *B. emersonii *seems to be an interesting model to study flagella-associated structures or functions.

Among the ESTs exclusively isolated from sporulating cells, we collected sequences associated to membrane transport, such as syntaxin 71. Membrane fusion and vesicle rearrangement are crucial events in *B. emersonii *sporulation, when cytokinesis occurs. A set of core SNAREs is apparently sufficient to mediate most intracellular vesicle fusion events, although multicellular organisms would express additional SNARE proteins for specific functions associated with the body complexity [51]. Thus, proteins like syntaxin 71 are good candidates to function as additional SNARE proteins in the transition of unicellular multinucleated zoosporangia to zoospores during *B. emersonii *life cycle.

Other collected ESTs were unexpected, like those involved in specific metabolic pathways, such as sequences involved in conversion of propionate and histidine to glutamate. We hypothesize that alternative pathways leading to the use of amino acids and other substrates as carbon and nitrogen sources could have been lost in late-diverging fungi and retained in basal fungi.

Finally, a large number of sequences selected by the first approach were not classified in a known process, which suggests that other structures or biological processes not identified yet can be shared by *B. emersonii*, animals and plants.

## Methods

### *B. emersonii *EST database

All the information concerning *B. emersonii *ESTs, such as construction and nucleotide sequencing of cDNA libraries, removal of contaminant sequences, and the annotation process were previously described [14]. The sequences are public and can be obtained from National Center for Biotechnology Information (NCBI) EST database (dbEST) [52] [dbEST:CO961503 – CO978552] or at the *Blastocladiella emersonii *database (BeDB) in the project website [43].

### Approach 1. Database source and construction, and pipeline for sequence comparative analysis

**Figure 4:**
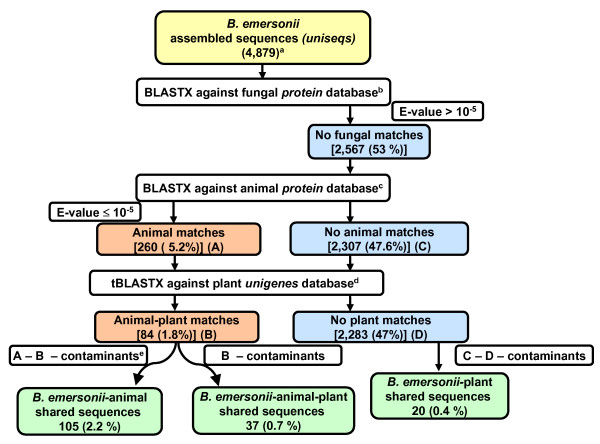
**Overview of the pipeline used in the EST comparative study, approach 1**. *B. emersonii uniseqs *were compared against animal and plant databases. ^a^Sequences from NCBI dbEST database [dbEST:CO961503 – CO978552]; ^b^sequences from nine fungal species; ^c^sequences from eight animal species; ^d^sequences from five plants species; ^e^sequences from species not included in our databases. See Methods section for details.

We constructed three databases *ad hoc*, representing fungal, animal and plant datasources, using the NCBI formatdb program to format them before carrying out local blast search. Protein sequences from eight distinct fungi and nine different animal species were downloaded from Genbank protein database and represent fungal and animal datasets, respectively. Considered species for fungal database were *Ustilago maydis, Saccharomyces cerevisiae, Schizosaccharomyces pombe, Candida albicans, Aspergillus nidulans, Neurospora crassa, Gibberella zeae *and *Magnaporthe grisea*. For animal database were chosen *Homo sapiens, Mus musculus, Tetraodon nigroviridis, Anopheles gambiae, Danio rerio, Rattus norvegicus, Xenopus leavis, Drosophila melanogaster *and *Caenorhabditis elegans*. A low proportion of plant protein data is found in public databanks, whereas EST collections have a more complete information set, even though redundant. Consequently, we based our plant database in unigene dataset (gene-oriented clusters of transcript sequences) from five plants (*Arabidopsis thaliana, Lycopersicon esculentum, Glycine max*, *Zea mays *and *Oryza sativa*) obtained also from Genbank. All data were collected between October 10 to 15, 2004, and final annotations and comparative analyses were updated up to April, 2006. Database sizes ranged from 40 to 138 million residues. When choosing species to incorporate into the datasets, the number of sequences deposited in Genbank and the biological representation into the group were considered. Searches throughout databases were carried out using the NCBI stand-alone blastall program. BLASTX and tBLASTX algorithms [53] were used for searching against protein databases and unigene databases, respectively. Linux tools and scripts were used to deal with data sets and blast outputs, and extract specific text/data lines of interest. The pipeline is summarized in Figure 4. Database sizes ranged from ~40 million to 138 million of amino acids and we used an Evalue ≤ 10^-5 ^as the cut-off to assign significance to best hit in the alignments. Final data to be analysed (indicated as "*B. emersonii*-animal shared sequences", "*B. emersonii*-plant shared sequences" and "*B. emersonii*-animal-plant shared sequences") were obtained after their filtration against species not included in our databases (fungi, plants and animals) to remove those sequences initially considered as not found in these groups, named as contaminants in this study. Hits found also in bacteria were specially checked for the presence of a poly A+ tail. Taking account that after the initial construction of the *ad hoc *databases several new fungal genomes became publicly available [6], we constructed two new fungal databases (protein and nucleotide bases) to proceed with the filtration. Data were downloaded from four of the several centres that have released genome sequences [6]: the Joint Genome Institute (JGI) [54], the Broad Institute [55] the University of Oklahoma [56] and the NCBI [57]. The complete list of species used to construct fungal databases is in Table 6. A local search against the new fungal bases was made using BLASTX or tBLASTX algorithms. We also used a client server program (blastcl3 program) and BLASTX or tBLASTX algorithms for remote search against non-redundant (nr) and dbEST-others databases from Genbank [58], respectively. Considering that databases at Genbank are larger than our ad hoc databases, we adjusted the E threshold to a less stringent value (~1 to 6E-4), maintaining S' constant (~50) and following the equation E = mn2^-S' ^[53]. Standalone blast and client server blast packages were downloaded from NCBI BLAST ftp site [59].

### Approach 2. Database source and construction, and pipeline for comparative sequence analysis

**Figure 5:**
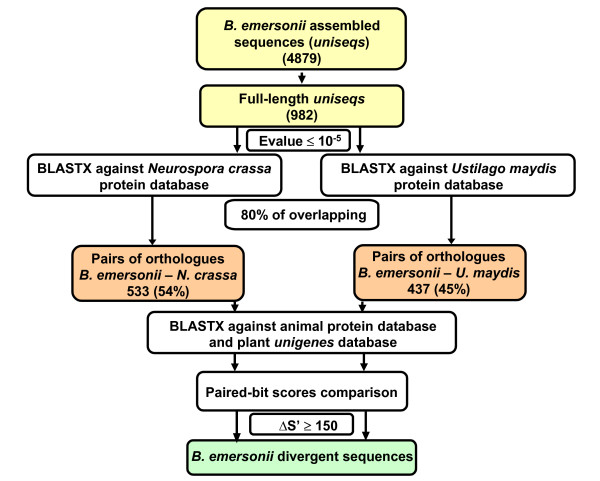
**Overview of the pipeline used in the EST comparative study, approach 2**. *B. emersonii *putative full-length *uniseqs *were compared against fungal protein sequences from *N. crassa *and *U. maydis*, and orthologous pairs were compared against animal and plant databases. See Methods section for details.

Two databases were constructed using *N. crassa *and *U. maydis *protein sequences downloaded from NCBI database. *N. crassa *and *U. maydis *were chosen in this study as representatives of ascomycetes and basidiomycetes with completely sequenced genomes, respectively. Putative orthologues of *B. emersonii *in *N. crassa *or *U. maydis *were obtained by comparing *B. emersonii *full-length sequences against the two fungal databases using BLASTX program. The pipeline is summarized in Figure 5. We chose not to proceed with a bidirectional best hit (BBH) comparison to select orthologous sequences because it could produce equivocal results, since *B. emersonii *transcriptome data are incomplete. Instead, we carried out a final manual revision of the resulting divergent sequences to exclude paralogues from our analysis. We accepted as homologues *B. emersonii *sequences that presented at least 80% of overlap with the corresponding protein sequences in *N. crassa *or *U. maydis*, and an Evalue ≤ 10^-5 ^as the cut-off to assign significance to best hit in the alignments. Full-length coding sequences were estimated as previously reported [14]. We based our analysis on the procedure adopted by Braun *et al*. [8] for comparing the amount of divergence. Pairs of putative orthologues from *N. crassa*/*U. maydis *and translated *B. emersonii *putative unique sequences were compared using BLASTP or tBLASTP against animal or plant databases, respectively, and the obtained *bit scores *(S') were recorded. A score difference equal or higher than 150 (ΔS' ≥ 150) was chosen to consider proteins as divergent. Divergent *bit scores *were re-evaluated by comparing them to other fungal vs. animal/plant scores to exclude divergences only proper to the two fungi initially considered.

### Sequence annotation

To assign a putative identification to *B. emersonii uniseqs*, we took into account BLASTX best-hit descriptions, or subsequent alignments with an Evalue below the assumed cut-off, resulting from sequence comparison against the *nr *and *dbEST-others *databases at NCBI. We also considered the biological process categories from Gene Ontology Consortium (GO) [60] attributed to *uniseqs *after comparison with sequences from curated databases (Swiss-Prot and TrEMBL) available at ExPASy proteomics server of the Swiss Institute of Bioinformatics (SIB) [61]. We maintained the GO structure we used in [14] for the classification of *B. emersonii *ESTs. This classification is available at [43]. However, GO classification is being upgraded continuously; upgrades can be checked in [60]. Other information sources were also consulted (mainly InterPro [62] and linked references, MIPS [63], Fantom3 [64] and FlyBase [65]) to refine the annotation.

## Authors' contributions

KFR designed the study, performed the first approach computational analysis, contributed with part of the data in the second approach analysis, and drafted the manuscript. RCG performed the second approach computational analysis, contributed with part of data in this second approach and helped to draft the manuscript figures. SLG participated in coordination and design of the study and helped to draft the manuscript. All authors read and approved the final manuscript.

## Figures and Tables

**Table 1:** Putative identification of 105 *B. emersonii*-animal shared sequences.

**CONTIG**	**PROCESS^a^**	**SUBPROCESS**	**DESCRIPTION**	**S^c^**	**ORGANISM**
BeAS318	cell growth^b^	transport	mannose-6-phosphate/insulin-like growth factor II receptor	62	**Animals**
BeE120N38E06	cell growth	microtubule-based process	Kinesin-associated protein 3	147	
BeZSPN12E10	cell growth	transport	proton-coupled dipeptide cotransporter	53	
BeE120N31C02	cell growth	transport	sperm-associated cation channel 2 isoform 1	71	
BeAS1332^1^	metabolism	L-methylmalonyl-CoA metabolism	**EC 5.1.99.1 Methylmalonyl-CoA-racemase**	204	
BeAS1259	metabolism		**EC 5.4.99.2 Methylmalonyl-CoA-mutase**	375	
BeZSPN11A04	signal transduction		inositol polyphosphate-4-phosphatase	63	
BeE120N37B06	signal transduction		guanylyl cyclase	77	
BeAS1273^1^	signal transduction		Arf-like 2 binding protein BART1	100	
BeE90N05E01^2^	development	sexual reproduction	sperm associated antigen 1 (predicted)	66	
BeE90N05C03	metabolism	protein amino acid phosphorylation	similar to CG32019-PA, isoform A	57	
BeE90N10F02	signal transduction	G-protein coupled receptor protein signaling pathway	Hypothetical protein CBG04044	56	
BeE120N07G08	unknown		LOC495042 protein	50	
BeE3018G09	unknown		similar to CG7382-PA	60	
BeE90N01G07	unknown		similar to ATP-binding cassette protein C12	60	
BeE90N07C01	unknown		ENSANGP00000002549 AG	59	
BeE90N08H06	unknown		unnamed protein product	50	
BeE90N12F11	unknown		similar to Myosin heavy chain	72	
BeE90N19F10^1^	unknown		similar to CG3313-PA	60	
BeE90N20B07	unknown		nonmuscle myosin heavy chain b	50	
BeE90N20E12	unknown		C20orf26	84	
BeE90N25E10	unknown		Origin recognition complex subunit 5	55	
BeG90N01F09	unknown		similar to KIAA0467 protein	55	
BeG90N13H11	unknown		ENSANGP00000021997	57	
BeE90N02H12	unknown		similar to Neurogenic locus notch homolog protein 1	69	
BeE90N19F09	unknown		similar to MEGF11 protein	64	
BeE120N02G09	unknown		unknown (WD repeat domain 34)*	63	
BeAS1968	unknown		unknown (leucine-rich)	60	
BeE60N20B11	unknown		Cc2-27, MGC83786*	53	
BeAS392	unknown		similar to RIKEN cDNA 5530601I19	74	
BeG30N12H05	unknown		C9orf119 protein	54	
BeAS991	unknown		Blu protein	107	
BeE60N03A11	unknown		unnamed protein product	100	
BeAS334	unknown		intraflagellar transport protein	71	
BeAS76	unknown		shippo	58	
BeE60N01H07	unknown		radial spokehead-like 1	70	
BeAS1855	unknown		unnamed protein product	85	
BeAS590	unknown		unnamed protein product*	94	
BeAS239	unknown		unnamed protein product	49	
BeAS1806	unknown		unnamed protein product	67	
BeAS1622	unknown		PHD finger protein 10*	62	
BeAS973	unknown		hypothetical protein*	63	
BeE120N38F02	unknown		predicted protein	58	
BeE60N12D05	unknown		ENSANGP00000021947	563	
BeAS1425	unknown		hypothetical protein	173	
BeE60N08G06	unknown		hypothetical protein*	158	
BeE60N16C07	unknown		hypothetical protein*	206	
BeE120N03F08	unknown		hypothetical protein	62	
BeAS898	unknown		ring finger protein 121 (RNF121)	95	
BeAS153	unknown		cortactin	97	
BeG30N15C05	unknown		K-Cl cotransporter	51	
BeE60N16H08	unknown		clusterin associated protein 1	93	
BeAS1786	unknown		SH3 and multiple ankyrin repeat*	96	
BeE120N34D07	unknown		axonemal dynein light chain p33	276	

BeAS107^2^	metabolism	proteolysis and peptidolysis	intraflagellar transport particle protein 140	124	**Animals and protists**
BeAS182^1^	metabolism	de novo pyrimidine base biosynthesis	involved in spermatogenesis	126	
BeE120N38D06	metabolism	regulation of transcription	RIKEN cDNA 4930506L13	137	
BeE90N20A03^1,3^	metabolism	GTP biosynthesis	similar to Ndpkz4 protein	100	
BeE90N03H03^2,3^	cell differentiation	spermatid development	sperm associated antigen 6 (SPAG6)	60	
BeE90N11E04^3,4^	development	morphogenesis	unnamed protein product	129	
BeE90N14D08^3,4^	response to stimulus	sensory perception	Unc-119 homolog	138	
BeE60N09G10^2^	cell growth	microtubule-based process	FLJ00203 protein	209	
BeAS1587	cell growth	microtubule nucleation	centromere protein J*	133	
BeAS279^1^	signal transduction		AKAP-associated sperm protein	91	
BeE120N06E01^2^	signal transduction	small GTPase mediated signal transduction	dynein 2 light intermediate chain*	139	
BeAS284^2^	signal transduction		similar to capillary morphogenesis protein-1	137	
BeAS1633^2^	unknown		spoke protein	152	
BeAS96^2^	unknown		protofilament ribbon protein	105	
BeE60N15D07^2^	unknown		IFT81*	155	
BeAS1699^2^	unknown		radial spokehead-like 1	116	
BeE90N01D06^1,3,4^	unknown		hypothetical protein, conserved	114	
BeG90N18C04^3^	unknown		Sfrs1 protein	54	
BeE90N05B01^3,4^	unknown		similar to CG17669-PA	124	
BeG60N12A12^4^	unknown		ENSANGP00000011450	76	
BeE90N13A07	unknown		hypothetical protein DDB0168470	68	
BeE90N15B10^3^	unknown		PREDICTED: hypothetical protein XP_787841	56	
BeE90N11E09^3^	unknown		similar to WD-repeat protein 56, partial	102	
BeE90N13H05^3,4^	unknown		similar to hypothetical protein	86	
BeE90N14C08^3,4^	unknown		similar to Nasopharyngeal epithelium specific protein 1, partial	91	
BeE90N18E06^3,4^	unknown		unnamed protein product	224	
BeE90N22D06^3,4^	unknown		Hypothetical protein LOC555400	99	
BeE90N01F04^3,4^	unknown		chromosome 21 ORF frame 59 variant	152	
BeAS78	unknown		PACRG (Parkin co-regulated gene)	286	
BeAS847	unknown		unc-93 homolog A	66	
BeAS380	unknown		C21orf59-like	136	
BeAS1625	unknown		zinc finger, MYND domain containing 12	176	
BeAS1475	unknown		unnamed protein product*	60	
BeE60N17F02	unknown		expressed protein	167	
BeAS1840	unknown		hypothetical protein*	55	
BeE60N15C02	unknown		RIKEN cDNA 9430097H08	163	
BeAS451	unknown		RIKEN cDNA 1700027N10	124	
BeAS240	unknown		CG1553-PB	103	
BeZSPN14C12^1^	unknown		Protein C21orf2	61	
BeAS1791	unknown		Putative adenylate kinase 7	124	
BeE60N04C06	unknown		signal recognition particle	81	
BeAS1698	unknown		hypothetical protein	58	

BeAS956	unknown		ubiquitin-like 3	55	**Animals (but also described in plants**
BeE60N06C03	unknown		probable katanin-like protein	70	

BeE30N11H04^1^	metabolism	histidine catabolism	**Hypothetical protein Amdhd1 protein**	216	**Animals and bacteria**
BeAS168Cd^5^	metabolism	L-methylmalonyl-CoA metabolism	**EC 6.4.1.3 Propionyl-CoA carboxilase**		
BeE30N13F08^2^	cell growth	cation transport	similar to sperm-associated cation channel 2 isoform 1	141	
BeE90N16A05	metabolism, signal transduction	regulation of transcription, two-component signal transduction system (phosphorelay)	putative two-component response regulator	167	
BeAS1512^1^	unknown		**EC 2.5.1.17 Adenosyltransferase**	195	
BeAS1143	unknown		CG4662-PB, LD23951p, unnamed	58	
BeAS509	unknown		Similar to RIKEN cDNA 2010311D03	95	

^1^Full-length sequences. ^2^Sequences associated with flagella. ^3^Matches with euglenozoos. ^4^Matches with ciliates. ^5^ESTs assembled in this *contig *were obtained from *B. emersonii *cells treated with cadmium (accession number DQ533709). ^a^Biological process, according to GO, assigned to the best hit in the specific database. ^b^cell growth means cell growth and/or maintenance. ^c^Bit score values obtained by searching against *nr *and *dbEST-others *(assigned as ESTs) databases from Genbank. *Genes presenting an Evalue between 10^-3 ^and 10^-5 ^against *ad hoc *fungal database. Proteins mentioned in the text are in bold

**Table 2:** Putative identification of 20 *B. emersonii*-plant shared sequences. See legend of Table 1 for details of the notes.

**CONTIG**	**PROCESS^a^**	**SUBPROCESS**	**DESCRIPTION**	**S^c^**	**ORGANISM**
BeAS808	metabolism	protein amino acid phosphorylation	**receptor protein kinase**	62	**Plants**
BeE90N21F06	metabolism	protein amino acid phosphorylation	**phytosulfokine receptor precursor**	82	
BeE90N13B04	signal transduction	two-component signal transduction system (phosphorelay)	**ethylene receptor CS-ETR2**	76	
BeAS1061	cell growth^b^	RNA-dependent DNA replication	unknown, putative reverse transcriptase*	70	
BeE90N13A06	unknown		ESTs	40	
BeE30N05D12	unknown		putative elicitor-responsive gene	51	
BeAS1555	unknown		putative elicitor-responsive gene	52	
BeZSPN17F09	unknown		ESTs*	60	
BeG90N16H10	unknown		ESTs	61	
BeAS1324	unknown		unknown	73	
BeAS1941	unknown		ESTs	56	

BeAS412	unknown		LMBR1 integral membrane family protein-like	169	**Plants and protists**
BeE120N27G09	unknown		LMBR1 integral membrane family protein	88	
BeE90N07H12	unknown		ESTs*	49	

BeAS412	cell growth	transport	putative syntaxin 71*	81	**Plants (but also described in animals and/or fungi)**
BeAS1606	unknown		putative isp4 protein*	55	
BeG60N07F02	unknown		ESTs*	49	
BeE90N18D12	unknown		putative DNA damage repair protein*	52	

BeZSPN13D02	metabolism	proteolysis and peptidolysis	ATP/GTP-binding site motif A (P-loop)	145	**Plants and bacteria**
BeE30N11G05	unknown		Putative transcription activator	74	

**Table 3:** Distribution of syntaxin family members in different main groups of organisms. The column with *B. emersonii *heading indicates the presence (yes) or absence (not found yet) of ESTs encoding the respective syntaxins in our libraries. SNARE proteins (including syntaxins) have been reclassified in two groups divided into five classes (see [41] and ref. there in). We have maintained the distribution according Sanderfoot *et al*. [24] to facilitate the comparison with our data.

**SUBFAMILIES of SYNTAXINS and their ORTHOLOGS**			
**PLANTS**	**ANIMALS**	**FUNGI (*S. cerevisiae*)**	***B. emersonii***

SYP1	Syn1	SSO1 and SSO2	Yes
SYP2	Syn7, Syn12 and Syn13	Pep12 and Vam3	Yes
SYP3	Syn5	Sed5p	Yes
SYP4	Syn16	Tlg2p	Yes
SYP5 and SYP6	Syn 8, Syn6 and Syn 10	Tlg1p	Yes
SYP7	NO ORTHOLOGS	NO ORTHOLOGS	**Yes**
SYP8	Syn18	Ufe1p	Not found yet

**Table 4:** Putative identification of 37 *B. emersonii*-animal-plant shared sequences. See legend of Table 1 for details of the notes.

**CONTIG**	**PROCESS^a^**	**SUBPROCESS**	**DESCRIPTION**	**S^c^**	**ORGANISM**
BeE30N11D12	metabolism	regulation of transcription	similar to PHD finger protein 16	61	**Animals and plants**
BeE30N11E01	metabolism	protein amino acid phosphorylation	receptor tyrosine kinase	54	
BeE30N16H02	metabolism, response to stimulus	electron transport, phototransduction	GA20503-PA	58	
BeE90N24F09	metabolism, signal transduction	protein amino acid phosphorylation, intracellular signaling cascade	CG3216-PB, isoform B	176	
BeAS682	unknown		hypothetical protein DDB0204189	51	
BeAS1783	unknown		similar to RIKEN cDNA 3110006P09	60	
BeAS701	unknown		similar to bicaudal-C	59	

BeAS1800	metabolism	histidine catabolism	**Probable urocanate hydratase (EC 4.2.1.49)**	307	**Animals, plants and protists**
BeAS1219	metabolism	proteolysis and peptidolysis	aminoacylase 1	134	
BeAS384^1^	metabolism	regulation of transcription, DNA-dependent	hypothetical protein DDB0188202	96	
BeE120N26E05	metabolism	nucleoside triphosphate	Nucleoside diphosphate kinase, putative*	80	
		biosynthesis			
BeE90N22D09	development	similar to transcription factor IIB	321		
BeE90N06A09	unknown		Zgc:101782	76	
BeE30N21F06	cell growth, metabolism	vesicle-mediated transport, lipid metabolism	similar to copine VIII	121	
BeE90N13D06^1^	response to stimulus	defense response	similar to Interferon-induced guanylate-binding protein	158	
BeE90N21E02	metabolism	cytoskeleton organization and biogenesis	LOC398504 protein	64	
BeAS315^1^	unknown		**ENSANGP00000015780**	105	
BeAS884^1^	unknown		**MTN3**	89	
BeAS1905	unknown		fiber protein Fb27	95	
BeAS891	unknown		similar to NN8-4AG*	134	
BeE120N08C01	unknown		similar to B9 protein	124	
BeE60N19G08^1^	unknown		rudimentary enhancer	75	

BeZSPN11C07^1^	cell growth, transport		**N-ethylmaleimide sensitive fusion protein attachment protein gamma**	70	**Animals and plants (but also described in fungi)**
BeZSPN17H06^1^	cell growth	transport	YfnA	86	
BeAS1770^1^	metabolism	intracellular protein transport	**Fructose-bisphosphate aldolase C**	416	
BeG30N01B09^1^	metabolism	nucleotide catabolism	5'-nucleotidase, cytosolic III	103	
BeAS1656^1^	metabolism	protein amino acid phosphorylation	RAC-gamma serine/threonine-protein kinase*	62	
BeAS1542	metabolism	electron transport	Acad8 protein*	92	
BeAS1889	metabolism	amino acid metabolism	glutamate dehydrogenase	193	
BeZSPN18F02^1^	unknown		**putative NEC1 Mtn3 family**	92	
BeE60N17G06	unknown		WD-repeat protein	71	

BeAS11^1^	metabolism, signal transduction	protein amino acid phosphorylation, intracellular signaling cascade	guanylyl cyclase	191	**Animals, plants and bacteria**
BeE90N18H07	metabolism	porphyrin biosynthesis	Putative oxygen-independent coproporphyrinogen III oxidase	106	
BeE90N21G11	signal transduction		putative membrane protein	151	
BeE90N24F08	unknown		Protein of unknown function UPF0061	124	
BeAS585	unknown		aminotransferase, putative	59	
BeAS64	unknown		hypothetical protein LOC554117*	138	

**Table 5:** Selected divergent and conserved orthologs from *B. emersonii *according to pairwise bit score plots. ΔS' = difference in *bit score *value. For each comparison plus and minus signs represent conserved and divergent *B. emersonii *sequences, respectively. Black circles identify a difference between *N. crassa *or *U. maydis *and *B. emersonii *scores when compared to animal or plant sequences (Nc A, Nc P, Um A and Um P, respectively). The database accession numbers below the circles correspond to sequences from *N. crassa*/*U. maydis *and animal/plant putative orthologs in each comparison. Data were selected from the plot in figure 3 and re-evaluated by comparison with other fungal vs. animal/plant scores. Database assembled sequences or accession numbers of *B. emersonii *and animal/plant putative orthologous sequences (same order as shown in the table) are: *B. emersonii*, [BeDB:BeAS1253, BeAS1274, BeAS745, and BeAS895]; animal data, [Genbank:DAA01331, AAB00075, AK060330, and EMBL: CAF97202]; plant data, [Genbank: AK062838, AK110624, and AK073448]

**DESCRIPTION**	**Δ S**	**NcA**	**NcP**	**UmA**	**UmP**
		●	●		
Rbj-like protein	-198	Genbank: EAA33910	Genbank: EAA33910		
	-193	Genbank: AAH45014	Genbank: AB018117		
		●		●	
elongation factor 1	-407	Genbank: EAA35632		Genbank: EAK82108	
alpha long form	-416	Genbank: AAB00075		PRF:2021264A	
			●		●
ADP/ATP translocase	-186		PIR:XWNC		Genbank: EAK82103
	-324		DDBJ: AK073448		DDBJ: AK073448
		●		●	
2-amino-3-carboxymuconate-	231	Genbank: EAA30585		Genbank: EAK85652	
6-semialdehyde decarboxylase	264	EMBL: CAF97202		RefSeq:NM_134372	

**Table 6:** Fungal expressed sequences used for constructing the two *ad hoc *fungal databases. Two new protein and nucleotide databases were used to filter out sequences not previously matched with fungal data belonging to the original fungal database. The nucleotide database included only unigenes, ESTs and mitochondrial sequences.

**Species/Strain**	**Data**	**Lineage**	**Sequencing/Source center**
*Ajellomyces capsulatus*	protein	Ascomycota/Eurotiomycetes	NCBI
*Aspergillus flavus*	protein	Ascomycota/Eurotiomycetes	NCBI
*Aspergillus fumigatus Af293*	protein	Ascomycota/Eurotiomycetes	FC/JGI
*Aspergillus nidulans FGSC A4*	protein	Ascomycota/Eurotiomycetes	Broad Institute/JGI
*Botrytis cinerea*	protein	Ascomycota/Leotiomycetes	Broad Institute
*Candida glabrata CBS138*	protein	Ascomycota/Saccharomycetes	Institut Pasteur/JGI
*Candida guillermondii*	protein	Ascomycota/Saccharomycetes	Broad Institute
*Candida lusitaniae*	protein	Ascomycota/Saccharomycetes	Broad Institute
*Chaetomium globosum*	protein	Ascomycota/Sordariomycetes	Broad Institute
*Coccidioides immitis*	protein	Ascomycota/Eurotiomycetes	Broad Institute
*Coprinus cinereus*	protein	Basidiomycota/Homobasidiomycetes	Broad Institute
*Cryptococcus neoformans H99*	protein	Basidiomycota/Heterobasidiomycetes	Broad Institute
*Cryptococcus neoformans JEC21*	protein	Basidiomycota/Heterobasidiomycetes	TIGR/JGI
*Debaryomyces hansenii CBS767*	protein	Ascomycota/Saccharomycetes	CNRS, Genoscope/JGI
*Encephalitozoon cuniculi GB-M*	protein	Microsporidia	Genoscope, Univ. Blaise Pascal/JGI
*Eremothecium gossypii*	protein	Ascomycota/Saccharomycetes	Basel Univ., Syngenta AG/JGI
*Fusarium graminearum*	protein	Ascomycota/Sordariomycetes	Broad Institute
*Gibberella zeae PH-1*	protein	Ascomycota/Sordariomycetes	International Consortium/JGI
*Kluyveromyces lactis NRRL Y-MHO*	protein	Ascomycota/Saccharomycetes	Univ. Claude Bernard, Genoscope, Institut Pasteur/JGI
*Magnaporthe grisea 70–15*	protein	Ascomycota/Sordariomycetes	Broad Institute/JGI
*Nectria haematococca*	protein	Ascomycota/Sordariomycetes	Joint Genome Institute
*Neurospora crassa*	protein	Ascomycota/Sordariomycetes	Broad Institute
*Phanerochaete crysosporium*	protein	Basidiomycota/Homobasidiomycetes	Joint Genome Institute
*Pichia stipitis*	protein	Ascomycota/Saccharomycetes	Joint Genome Institute
*Rhizopus oryzae*	protein	Zigomycota/Zygomycetes	Broad Institute
*Saccharomyces cerevisiae*	protein	Ascomycota/Saccharomycetes	International Consortium/JGI
*Schizosaccharomyces pombe 972 h*	protein	Ascomycota/Schizosaccharomycetes	Sanger Institute, Cold Spring Harbor Laboratory/JGI
*Sclerotinia sclerotiorum*	protein	Ascomycota/Leotiomycetes	Broad Institute
*Stagonospora nodorum*	protein	Ascomycota/Dothideomycetes	Broad Institute
*Trichoderma reseei*	protein	Ascomycota/Sordariomycetes	Joint Genome Institute
*Ustilago maydis*	protein	Basidiomycota/Ustilagomycetes	Broad Institute
*Yarrowia lipolytica CLIB122*	protein	Ascomycota/Saccharomycetes	CNRS, Genoscope/JGI

*Ajellomyces capsulatus*	ESTs	Ascomycota/Eurotiomycetes	Washington University/NCBI
*Aspergillus flavus*	unigene	Ascomycota/Eurotiomycetes	University of Oklahoma
*Botrytis cinerea*	mitochondrial	Ascomycota/Leotiomycetes	Broad Institute
*Candida tropicalis*	mitochondrial	Ascomycota/Saccharomycetes	Broad Institute
*Coccidioides immitis*	mitochondrial	Ascomycota/Eurotiomycetes	Broad Institute
*Coprinus cinereus*	ESTs	Basidiomycota/Homobasidiomycetes	Patricia Pukkila, Univ. North Carolina Chapel/Broad Institute
*Coprinus cinereus*	unigene	Basidiomycota/Homobasidiomycetes	University of Oklahoma
*Cryptococcus neoformans 184A*	ESTs	Basidiomycota/Heterobasidiomycetes	University of Oklahoma
*Cryptococcus neoformans B3501*	ESTs	Basidiomycota/Heterobasidiomycetes	University of Oklahoma
*Cryptococcus neoformans H99*	ESTs	Basidiomycota/Heterobasidiomycetes	University of Oklahoma
*Fusarium sporotrichiodes*	unigene	Ascomycota/Sordariomycetes	University of Oklahoma
*Fusarium verticillioides*	mitochondrial	Ascomycota/Sordariomycetes	Broad Institute
*Histoplasma capsulatum*	mitochondrial	Ascomycota/Eurotiomycetes	Broad Institute
*Laccaria sp*.	ESTs	Basidiomycota/Homobasidiomycetes	Joint Genome Institute
*Magnaporthe grisea*	mitochondrial	Ascomycota/Sordariomycetes	Broad Institute
*Neurospora crassa*	mitochondrial	Ascomycota/Sordariomycetes	Broad Institute
*Neurospora crassa*	unigene	Ascomycota/Sordariomycetes	University of Oklahoma
*Rhizopus oryzae*	mitochondrial	Zigomycota/Zygomycetes	Broad Institute
*Sclerotinia sclerotiorum*	mitochondrial	Ascomycota/Leotiomycetes	Broad Institute
*Uncinocarpus reesii*	mitochondrial	Ascomycota/Eurotiomycetes	Broad Institute
*Ustilago maydis*	mitochondrial	Basidiomycota/Ustilagomycetes	Broad Institute
